# Estimation and comparison of the effective dose and lifetime attributable risk of thyroid cancer between males and females in routine head computed tomography scans: a multicentre study

**DOI:** 10.1002/jmrs.752

**Published:** 2024-01-12

**Authors:** Daryoush Khoramian, Mohammad Haghparast, Adnan Honardari, Ebrahim Nouri, Esmail Ranjbar, Razagh Abedi‐Friouzjah, Shiva Zarifi, Choirul Anam, Milad Najafzadeh, Mahdieh Afkhami‐Ardakni

**Affiliations:** ^1^ The Advocate Centre for Clinical Research Ayatollah Yasrebi Hospital Kashan Iran; ^2^ Department of Medical Physics, School of Medicine Iran University of Medical Sciences Tehran Iran; ^3^ Department of Radiology, Faculty of Para‐Medicine Hormozgan University of Medical Sciences Bandar‐Abbas Iran; ^4^ Department of Anatomy and Cell Biology, School of Medicine Mashhad University of Medical Sciences Mashhad Iran; ^5^ Cellular and Molecular Research Centre Yasuj University of Medical Sciences Yasuj Iran; ^6^ Radiation Oncology Department, Imam Reza Hospital, Faculty of Medicine Mashhad University of Medical Sciences Mashhad Iran; ^7^ Department of Physics, Faculty of Mathematics and Sciences Diponegoro University Semarang Indonesia; ^8^ Department and Research Centre of Medical Physics Mashhad University of Medical Science Mashhad Iran

**Keywords:** CT scan, effective dose, head, lifetime attributable risk, thyroid dose

## Abstract

**Introduction:**

A significant number of head computed tomography (CT) scans are performed annually. However, due to the close proximity of the thyroid gland to the radiation field, this procedure can expose the gland to ionising radiation. Consequently, this study aimed to estimate organ dose, effective dose (ED) and lifetime attributable risk (LAR) of thyroid cancer from head CT scans in adults.

**Methods:**

Head CT scans of 74 patients (38 males and 36 females) were collected using three different CT scanners. Age, sex, and scanning parameters, including scan length, tube current–time product (mAs), pitch, CT dose index, and dose‐length product (DLP) were collected. CT‐Expo software was used to calculate thyroid dose and ED for each patient based on scan parameters. LARs were subsequently computed using the methodology presented in the Biologic Effects of Ionizing Radiation (BEIR) Phase VII report.

**Results:**

Although the mean thyroid organ dose (2.66 ± 1.03 mGy) and ED (1.6 ± 0.4 mSv) were slightly higher in females, these differences were not statistically significant compared to males (mean thyroid dose, 2.52 ± 1.31 mGy; mean ED, 1.5 ± 0.4 mSv). Conversely, there was a significant difference between the mean thyroid LAR of females (0.91 ± 1.35) and males (0.20136 ± 0.29) (*P* = 0.001). However, the influencing parameters were virtually identical for both groups.

**Conclusions:**

The study's results indicate that females have a higher LAR than males, which can be attributed to higher radiation sensitivity of the thyroid in females. Thus, additional care in the choice of scan parameters and irradiated scan field for female patients is recommended.

## Graphical abstract

The thyroid's effective dose increases with increasing DLP values. A decreasing trend in LAR values is observed with increasing age. The thyroid's mean effective dose and LAR in females are higher than in males because of the higher radiosensitivity of the thyroid gland in females.

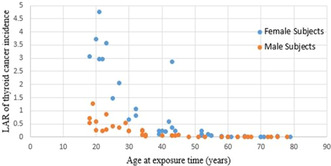

## Introduction

Computed tomography (CT) is a tomographic imaging technique that reconstructs images using X‐ray transmission and computer algorithms. New CT scanners, including wide detectors, dual‐energy and dual‐source scanners, have improved image quality parameters such as contrast and temporal resolution.^1^ Recent studies show that the number of CT procedures performed in the US has increased to around 84 million annually, with 18.9% of these being head CT scans.^2^ Additionally, CT is responsible for over 44% of the global collective effective dose (ED) equivalent based on the ICRP 103 recommendations,^3^ with head CT contributing 5.9%. Consequently, despite its extensive clinical application, there are growing concerns about the radiation dose imposed on patients during head CT procedures.

In a head CT scan, the thyroid dose results from scattered radiation. Although considered low, it still poses a non‐negligible risk of cancer induction. Exposure to ionising radiation is a significant risk factor for thyroid cancer of which one‐third is malignant.^4^ The incidence rate of thyroid cancer is higher in females than males, with estimates of 4.7 and 1.5 cases per 100,000 individuals for females and males, respectively.^4^ Younger patients, especially females, are at a higher risk of radiation‐induced cancer compared to older patients.^5^

The estimation of the thyroid's ED and the risk of radiation‐induced cancer has been studied mostly in children and phantoms using various CT imaging protocols.^5^, ^6^, ^7^, ^8^, ^9^ As a result, the thyroid dose was only evaluated during paediatric head CT procedures, adult patients undergoing neck CT procedures, and chest CT procedures using a chest phantom, but not during adult head imaging.^10^, ^11^ Moreover, studies on the thyroid's ED and lifetime attributable risk (LAR) for both sexes (male and female) have received less attention.^12^, ^13^, ^14^, ^15^ Only a few studies have evaluated the risk of thyroid cancer from head CT scans in adults of both sexes using the risk model recommended by the Committee on the Biological Effects of Ionizing Radiation (BEIR).^12^, ^15^, ^16^

Maziar et al.^17^ assessed the mean ED of thyroid radiation in adults who underwent head CT scans. A prospective study conducted in Nigeria found that females and children have a significantly higher risk of thyroid cancer than males.^18^ Similarly, Kiani et al.^19^ found that female paediatric patients have a greater risk of developing thyroid cancer than male paediatric patients. Intriguingly, a study conducted in Iran aimed to estimate the LAR of thyroid cancer in paediatric patients who underwent CT scans of the head scan and found an increase in the average LAR of thyroid cancer in females compared with males.^20^ Another study by Sulieman et al.^5^ demonstrated that female patients who underwent chest CT scans have an increased risk of thyroid cancer.

Estimating and comparing the ED and LAR of thyroid cancer in adults who undergo head CT procedures can be useful, considering the associated limitations. Therefore, this study aims to investigate and compare the ED and LAR of thyroid cancer between male and female patients who undergo routine head CT scans.

## Materials and Methods

### Data collection

This prospective study was conducted between September and November 2019. Initially, all adult head CT examinations of trauma and neurosurgical patients performed at our institution were evaluated. Exclusion criteria were paediatric patients (under 18 years), patients with special conditions, including cancer and enhanced CT protocols using contrast agents. Additionally, we checked the picture archiving and communication system (PACS) to ensure that dose information was available for each patient. The CT scans were performed using Toshiba Alexion 16 slice (TA) (Toshiba Medical Systems, Nasu, Japan), Philips Ingenuity 16 slice (PI) (Philips Medical Systems, Best, the Netherlands), and Siemens Somatom Emotion 16 slice (SSE) (Siemens AG, Erlangen, Germany). Table 1 presents the features of each scanner.

**Table 1:** Features of the scanners used in this study.

Scanner	Vendor	X‐ray tube	Gantry aperture (cm)	Focal spot (mm)	Total filtration (mm Al equivalent)	Detector rows	Number of detectors in the *z*‐axis	Tube current range (step)
Siemens Somatom Emotion	Siemens	Siemens Dura 422MV	70	0.8 × 0.5, 0.8 × 0.7	6	16 Ultra‐Fast Ceramic	24	20–345 (1 mA)
Philips Ingenuity Flex	Philips	Philips MRC	70	1 × 1, 0.5 × 1	>5.5	16 Solid‐state with TACH	24	20–500 (1 mA)
Toshiba Alexion	Toshiba	Toshiba Helicool (CXB‐400C)	72	1.6 × 1.4, 0.9 × 0.7	>2.5	16 Solid‐state	28	10–300 (10 mA)

Data obtained from the head CT examination of each patient included information such as age and sex, technical parameters such as the tube potential (kV), tube current (mA), rotation time (s), slice thickness (mm), acquisition collimation (mm), scan length (mm) and pitch. Exposure data like volumetric CT dose index (CTDI_vol_) and dose‐length product (DLP) were extracted from the PACS system For the scan length, First patient data and their dose reports were loaded from the PACS system and then the scan length was measured using the ratio of DLP to CTDIvol. During all scans, tube current modulation systems such as CARE DOSE 4D in the Siemens scanner, iDOSE4 in the Philips scanner, and SURE Exposure in the Toshiba scanner were deactivated.

### Thyroid organ dose and ED estimation

Several methods have been proposed to estimate the radiation dose to organs and ED in CT scans. In this study, tissue weighting coefficients recommended by the International Commission on Radiological Protection (ICRP) were used to estimate the organ dose and ED.^3^ To this end, CT dosimetry software based on the Monte Carlo method, called CT‐Expo software (version 2.6.1, Medizinische Hochschule, Hannover, Germany) was employed, which calculates the dose values specifically for different age and gender groups, including adult males, females, children and infants. This software has been benchmarked and used by various researchers for CT dose estimation,^21^, ^22^ and the accuracy of its dosimetry data has been confirmed.^22^, ^23^ For the current study, the doses using adult male and female phantoms were estimated. The software includes four model phantoms for dose estimation in paediatrics: an adult male phantom (170 cm height and 70 kg weight), an adult female phantom (160 cm height and 60 kg weight), a child phantom (115 cm height and 22 kg weight), and an infant phantom. Using the ICRP‐103 tissue weighting factor, the CT‐Expo software can estimate the thyroid organ dose and ED.

### LAR estimation

**Figure 1:**
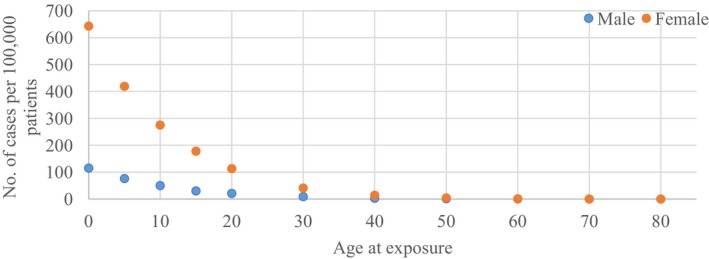
The LAR of cancer incidents for thyroid cancer according to the BEIR VII. Data are presented as the number of cases per 100,000 patients for a single dose of 0.1 Gy. The permission was obtained to reproduce this figure from National Academies Press. BEIR, Biological Effect of Ionising Radiation.

The LAR of thyroid cancer incidence was estimated using the model recommended by the BEIR VII report.^16^ The model is based on a linear no‐threshold approach. If an individual is exposed to a certain dose (*D*) at a particular age (*e*), then the LAR can be calculated using equation (1):

(1) $$\text{LAR}\left(D,e\right)=\sum_{a}M\left(D,e,a\right)\frac{S\left(a\right)}{S\left(e\right)}$$

where *a* denotes the attained age (in years), *M* (*D*, *e*, *a*) represents the excess relative risk (ERR), which is the rate of disease in an exposed population minus the rate of disease in an unexposed population, *S*(*a*) is the probability of survival until age *a*, and *S*(*a*)/*S*(*e*) signifies the probability of survival to age *a* given survival to age *e*. Table 12D‐1 from the BEIR VII report was used to calculate the LAR of thyroid cancer incidence. These data were extrapolated to obtain the required information. Figure 1 provides a baseline for our calculations, showing the LAR for thyroid cancer in both sexes.^16^

### Effect of the scan parameters on CTDI_vol_ and LAR

The scan parameters used in this study included mAs, kVp, scan length, beam width and pitch (Table 3). Tube current or the product of tube current and scan time (mAs) in CT scans determines the amount of X‐ray radiation generated in the tube. A linear relationship exists between tube current and CTDI_vol_, meaning that an increase in the mA or mAs can lead to a comparable percentage increase in the dose.^24^ LAR is strictly dependent on the radiation dose^16^ (Eq. 1), and any parameter that enhances the dose also increases the LAR.^16^ Peak kilovoltage (kVp) is the potential difference between the anode and cathode, which accelerates electrons towards the anode to produce X‐ray radiation. A specific relationship exists between kVp and radiation dose.^24^ The CTDIvol is approximately proportional to the square of kVp, and reducing the kVp from 120 to 100 could reduce the CTDI by a factor of 33%.^25^ Reducing the CTDI_vol_ also decreases the LAR.

The beam width is the total width of the X‐ray beam output that enters the patient's body, defined in the isocentre of the CT scanner.^26^ Decreasing this parameter while keeping other factors constant can lead to a decrease in CDTI_vol_ and LAR.^26^ Pitch is the table movement ratio per one gantry rotation to the total beam width.^24^ Pitch has an inverse relationship with CTDI_vol_. Thus, a higher pitch value corresponds to a lower CTDI_vol_ and LAR, and vice versa.^27^ Finally, the total received dose by the patient for each type of scan is influenced by the length of the scanned region – a longer scan length results in a higher patient DLP and LAR.^27^

### Statistical analyses

All statistical analyses were performed through SPSS v.16.0 (IBM Corp., Armonk, NY, USA). The Kolmogorov–Smirnov test was used to assess the normality of the data distribution. The Mann–Whitney U‐test and independent two‐sample *t*‐test were used to compare parameters between the male and female groups. Subsequently, linear regression was performed to investigate the strength of the correlation between the two variables. A *P*‐value < 0.05 was considered statistically significant for all the statistical analyses.

## Results

The sample size for the study was selected based on a G*power analysis and a previous study by Sulieman et al.,^5^ where 60 samples were used. To obtain more reliable results, a sample size of >60 was selected. The power analysis results confirmed that with a sample size of 74, differences between the two groups (with Cohen's effect size = 0.66–0.8) can be statistically significant at the 0.05 level with a power of 0.8. A total of 74 patients (38 males and 36 females) who had undergone non‐contrast head CT scans were included in the study. Seven patients were excluded from the sample as their CT scan did not have a dose report page in the PACS.

The mean ± standard deviation (SD) age of male patients was 43 ± 19 years (range, 18–78 years), while that of female patients was 46 ± 17 years (range, 18–79 years). There was no statistically significant difference in age between the male and female groups (*P*‐value = 0.61). Table 2 provides a list of all scanners, patients' sex distributions, and scan parameters used for each scanner. All examinations were conducted in helical mode, except for the SSE scanner, which uses the conventional mode as the first choice for head scans in our imaging department. A total of 28 patients underwent scanning on the SSE 16‐slice scanner at 110 kVp, while 46 patients were scanned on TA 16‐slice and PI 16‐slice scanners at 120 kVp (Table 2). The scan parameters for both male and female groups are summarised in Table 3.

**Table 2:** List of scanners, patients' sex distributions and scan parameters used for each scanner.

Scanner	Patient sex		Tube current–time (mAs)	Kilovoltage peak (kVp)	Scan length (cm)	Scan mode	Rotation times (s)	Beam width
	Male	Female						
Siemens Somatom Emotion	15	13	205.04 ± 55.65	110	13.18 ± 1.6	Axial (pitch = 1)	1	10
Philips Ingenuity Flex	12	15	188.39 ± 4.86	120	15.8 ± 1.7	Helical (pitch = 0.563)	0.5	10
Toshiba Alexion	11	8	160 ± 0	120	14.24 ± 0.98	Helical (pitch = 1)	1.5	9.6

Values are expressed as mean ± standard deviation.

**Table 3:** Comparison of scan parameters used for the head CT protocols.

Subjects	Tube current–time (mAs)	Kilovoltage peak (kVp)	Scan length (cm)	Helical pitch	Beam width (mm)	Rotation time (s)
Females	195.16 ± 52 (190, 160–199)	116.28 ± 4.9 (120, 110–120)	15.73 ± 3.9 (14.6, 10.8–16.1)	0.87 ± 0.31 (1, 0.563–1)	9.91 ± 0.17 (10, 9.6–10)	0.9 ± 0.39 (1, 0.5–1.5)
Males	186.1 ± 37.4 (190, 160–220)	116.15 ± 4.93 (120, 110–120)	14.6 ± 1.57 (14.4, 10.7–17.5)	0.77 ± 0.38 (1, 0.563–1)	9.88 ± 0.18 (10, 9.6–10)	0.98 ± 0.39 (1, 0.5–1.5)
*P*‐value	0.48	0.77	0.73	0.28	0.52	0.36

Values are represented as mean ± standard deviation, median and range.

While there were no significant differences between the groups in the scanning parameters, the mean (±SD) values of mAs were 186.1 ± 37.4 and 195.16 ± 52 for the males and females, respectively (ranging from 160 to 220 for the females and 160 to 198 for the males) (Table 3). The mean (±SD) scan length for males and females were 15.73 ± 3.9 and 14.6 ± 1.57, respectively (Table 3). The scan length was from the top of the head to the C1 lamina, For the beam width, the mean value for the male groups was 9.88 ± 0.18 mm and for female groups was 9.91 ± 0.17 mm (ranged from 9.6 to 10 mm), respectively. The rotation times ranged from 0.5 to 1.5 s. Two different pitch values of 0.5 and 1 were used for helical scan.

**Figure 2:**
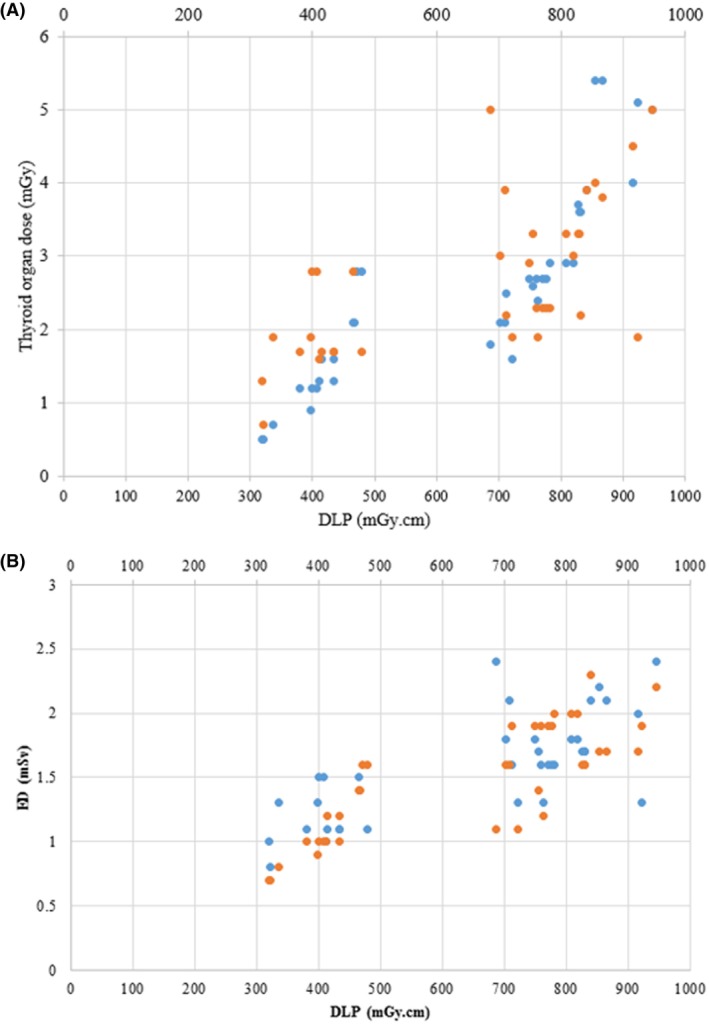
(A) Variation of thyroid organ dose versus DLP range (as evident, the thyroid organ dose increases with increase in DLP). Blue dots: Female subjects, *R* ^2^ = 0.76, Orange dots = Male subjects, *R* ^2^ = 0.84. DLP, dose length product. (B) Variations of ED versus DLP range (as evident, the ED increases with increase in DLP). DLP, dose length product; ED, effective dose. Blue dots: Female subjects, *R* ^2^ = 0.84, Orange dots = Male subjects, *R* ^2^ = 0.81.

Table 4 displays the differences in thyroid dose between the two scanning modes (axial and helical) for both males and females. The results show that the thyroid dose in helical mode was significantly higher than in axial mode (*P* < 0.001 for males and females) (Table 4). The dose difference between helical and axial scans was 1.23 and 65% for females and 1.78 and 122% for males. Table 5 shows a comparison of CTDI_vol_, DLP, thyroid organ dose, ED and LAR for the two groups. The median values for the females were 53.1 ± 11.36 for CTDI_vol_ and 700 ± 188.7 for DLP. For the males, the median CTDI_vol_ value was 48.55 ± 10.4 mGy, and the median DLP was 717.2 ± 203 mGy.cm. The thyroid organ dose was plotted against DLPs for both groups (Fig. 2A). As DLP increased, the patient's thyroid dose also increased. The coefficient of determination (R‐squared) for females and males was 0.76 and 0.84, respectively. This indicates the existence of a positive and significant correlation between these parameters in the two groups (*P* < 0.001, *R* ^2^ > 0).

**Table 4:** Comparison of the thyroid dose between the helical and axial scanning modes in the head CT.

Subjects	Thyroid dose (mGy)		Difference	Percentage difference	*P*‐value
	Scanning modes				
	Helical (*a*)	Axial (*b*)			
				(*a* − *b*)/*b* × 100	
Female	3.1 ± 0.96	1.87 ± 0.61	1.23	65	<0.001
Male	3.23 ± 1.13	1.45 ± 0.73	1.78	122	<0.001

**Table 5:** Mean and SD values of the thyroid organ dose, ED and LAR.

Subjects	CTDI_vol_ (mGy)	DLP* (mGy × cm)	Organ dose (mGy)	ED (mSv)	LAR (per 100,000 people)
Females	44.84 ± 11.36 (53.1, 29–53.9)	635.4 ± 188.7 (700.1, 332–867.6)	2.66 ± 1.03 (2.3, 0.7–5)	1.6 ± 0.4 (1.6, 1.1–2.4)	0.91 ± 1.35 (0.23, 0.00087–4.761)
Males	42.69 ± 10.46 (48.55, 30.1–53.9)	644.6 ± 203.0 (717.2, 319.8–946.7)	2.52 ± 1.31 (2.55, 0.5–5.4)	1.5 ± 0.4 (1.6, 0.7–2.3)	0.2 ± 0.29 (0.064, 0.0002–1.3)
*P* value	0.07	0.57	0.43	0.35	0.001

Values are represented as mean ± standard deviation, median and range values. CTDI_vol_, volume computed tomography dose index; DLP, dose length product; ED, effective dose; LAR, lifetime attributable risk.

* DLP, dose length product, is the product of the CTDIvol and the scan length of a group of scans.

**Figure 3:**
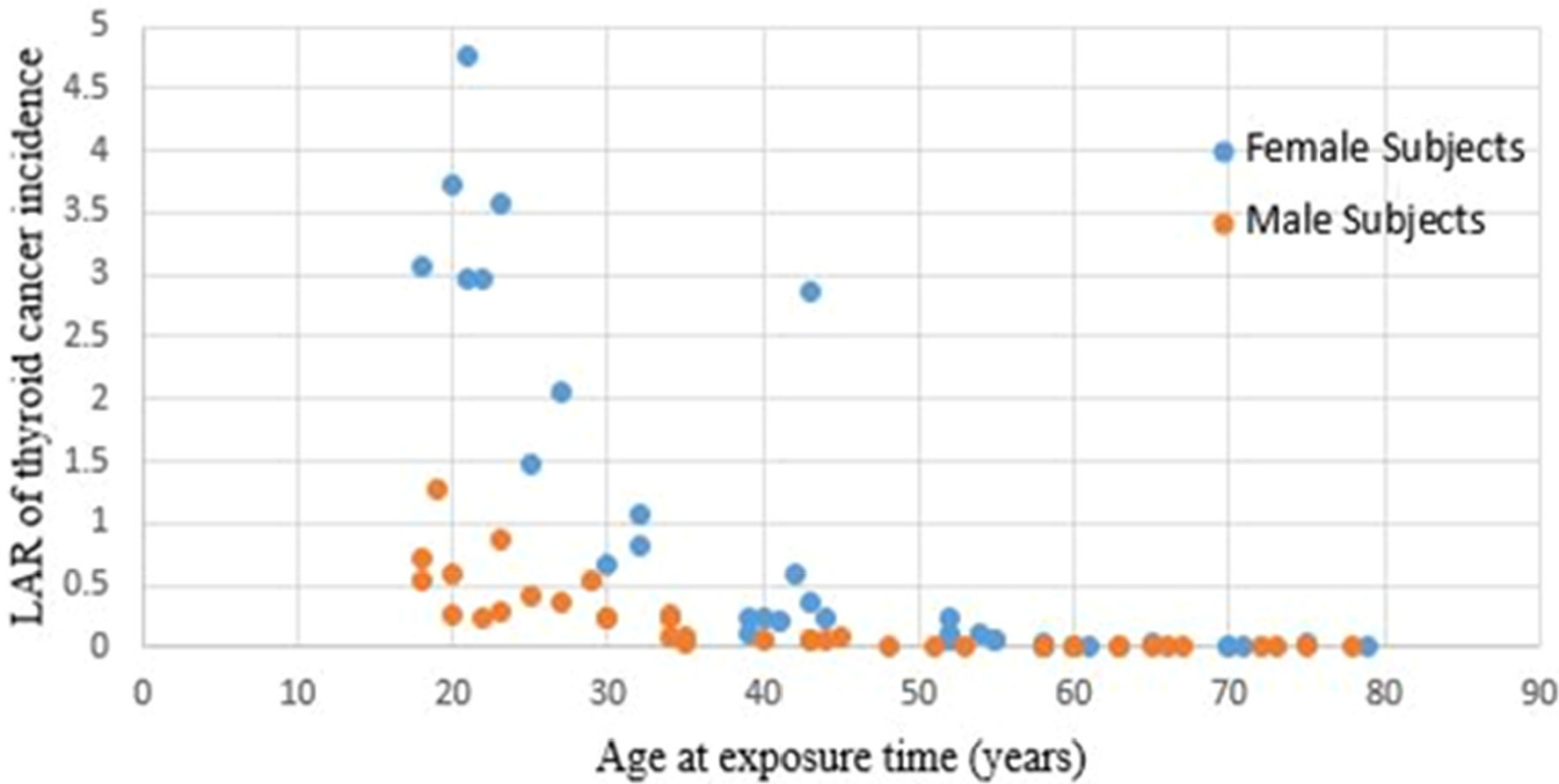
Variations of LAR versus the age range (as evident, the LAR decreases once the age increases). LAR, lifetime attributable risk.

The distribution of ED against DLPs is shown in Figure 2B. Similar to the thyroid organ dose, ED increased as DLPs increased. The coefficient of determination (R‐squared) was 0.84 and 0.81 for females and males, respectively. Figure 3 shows the variations in LAR by age at the time of exposure for both males and females. As expected, a decreasing pattern in LAR with age was observed. All values, including the age, tube current–time product, scan length, CTDI_vol_, DLP, ED, thyroid dose and LAR, were tested for normality. The tube current–time, CTDI_vol_, DLP, thyroid dose, and LAR (both groups) were not normally distributed among the data.

The statistical analyses revealed that there were no significant differences (*P* > 0.05) in most parameters between the male and female groups. However, it was observed that the female group had a significantly higher LAR compared to the male group (*P*‐value = 0.001 < 0.05, Table 5).

## Discussion

The use of CT scans has become increasingly prevalent in the medical field, particularly for diagnostic purposes. Head CT imaging, among other types of CT imaging, has been widely employed as a diagnostic tool for brain injuries. According to Asadinezhad et al.,^28^ 77.03% of all CT scans conducted in Iran were head CT scans.

### CTDI_vol_ and DLP parameters for head CT imaging

Previous researches have demonstrated that undergoing head CT scans can increase the risk of developing thyroid cancer.^4^, ^29^ Therefore, it is crucial to justify and optimise the use of head CT examinations. One way to optimise these scans is by monitoring the radiation exposure to the thyroid gland caused by head CT scans and establishing diagnostic reference levels (DRLs) to guide efforts towards reducing radiation doses in CT exams. DRLs play a critical role in managing radiation doses in CT scans and serve as a guideline to minimise radiation exposure in CT scans.

There are two national dose reference levels (NDRLs) for CT imaging parameters in Iran. According to Sohrabi et al.,^30^ the NDRLs for median CTDI_vol_ and DLP for brain CT imaging are 58 ± 28.84 mGy and 750 ± 339.37 mGy.cm, respectively. However, Asadinezhad et al.^28^ reported different values for the median CTDI_vol_ and DLP, which were 49.85 ± 17.73 mGy.cm and 1161 ± 426.91 mGy.cm, respectively. In this study, the median CTDIvol and DLP values were found to be 53.1 mGy and 700 mGy.cm for females and 48.55 mGy and 717 mGy.cm for males (Table 5), which are almost identical to the values reported by Asadinezhad et al.^28^ (CTDIvol difference = 3.2%, DLP difference = 3.2%).Conversely they are largely different from those reported by Sohrabi et al.^30^ (CTDIvol difference = 11.2%, DLP difference = 40%). The discrepancy between the obtained values and the NDRLs may be due to the fact that CTDI_vol_ depends on the scan parameters such as mAs, kVp, beam width, pitch and the operator's skill, including the improper positioning of patients in the isocentre and scanner model.^26^

The study conducted by Asadinezhad et al.^28^ lacked details on the scan parameters, so the results were compared with the study conducted by Sohrabi et al.^30^ The kVps used in this study had a mean of 116 and a range of 110–120, and the beam width had a mean of 9.91 and a range of 9.6–10 (Table 3). These values were lower than those used in Sohrabi's study, with a mean of 117 and a range of 120–140 for kVp.^30^ Another study indicated that increasing the kVp by 12% can increase CTDI_vol_ by up to 29%.^24^ Moreover, when the kVp is changed from 120 to 140 while keeping other parameters constant, the percentage of increase can be up to 37%.^31^, ^32^

This trend was also observed for the beam width and pitch. Sohrabi et al.^30^ utilised a larger mean beam width of 10.2 mm compared to our study's mean beam width of 9.89 mm, which ranged from 5 to 40 mm compared to our study's range of 9.6–10 mm. The mean mAs value in our study was 190.63, which was 18% higher than the mAs value used by Sohrabi et al.^30^ (160.3 mAs). It should be noted that Sohrabi et al.^30^ used a wider range of mAs (100–350 mAs) compared to our study (160–220 mAs). As mentioned in the previous section, there is a linear relationship between the CTDI_vol_ and mAs.

Another significant fact to consider is the type of scanner model used. In this study, all but one of the multi‐slice scanners used worked in the axial mode, and AEC was deactivated during all scans. Sohrabi et al., on the other hand, used a variety of scanner models, including both single‐slice and multi‐slice scanners. Another factor to consider is the proper positioning of patients in the isocentre.^28^, ^30^ Previous studies have shown that improper positioning can significantly increase the radiation dose in CT scans by up to 30%.^33^, ^34^

In both studies, the mean pitch value was almost identical, with a pitch difference of only 1%. However, the median DLP value was found to be lower in the current study than in the previous survey, with values of 6.6% and 40% respectively^28^, ^30^ (Table 5). The difference in DLP could be due to the higher scan length (29%) used in the present study, as compared to the study conducted by Sohrabi et al.^30^ This difference can be explained by the fact that DLP is a product of CTDI_vol_ and scan length. Therefore, reducing both the CTDI_vol_ and scan length can help in decreasing the value of DLP.

### Thyroid organ dose and ED

In contrast to the benefits of CT scans, concerns have been raised regarding the risks of this low‐radiation imaging method, particularly for radiosensitive organs exposed to radiation.

When CT scans are taken of the head, neck, and chest, the thyroid glands are given special attention because they are sensitive to radiation. Table 5 shows that the mean thyroid organ dose was slightly higher in females (2.66 ± 1.03 mGy) than in males (2.52 ± 1.31 mGy), which is consistent with previous studies.^13^, ^18^, ^35^ Tipnis et al.^36^ found that the effect of CTDI_vol_ on thyroid dose depended on tube potential, tube current, beam collimation and beam pitch. Since these parameters were not significantly different between the male and female groups (Table 3), there was no difference in the thyroid dose between the groups. However, the size of the phantom should also be considered. The neck length of an adult male phantom in the CT‐Expo software was 9 cm, while that of the female phantom was 8 cm.^37^ A smaller neck size increases the chance of scattered radiation reaching the thyroid, which may have contributed to the differences in thyroid dose between the male and female groups.

There was a similar trend observed for the ED in both male and female patients, with a slightly higher mean ED in females (Table 5). These results are consistent with previous studies.^15^ Furthermore, the thyroid organ dose was compared between axial and helical scans for both male and female patients, and it was found that there were significant differences between the two modes of scanning. Specifically, the helical scan resulted in a much higher radiation dose to the thyroid than the axial scan for both groups (*P* < 0.001, Table 4). The percentage difference between helical and axial scans was significant, with a 122% difference for males and a 65% difference for females. The presence of pitch in the helical scan causes over‐scanning, which creates more scattered radiation than the axial scan due to three factors: beam collimation, reconstructed slice thickness, and pitch. These findings are consistent with previous studies.^15^, ^38^

The correlation between the dose that the thyroid organ receives and the DLPs and ED values showed a linear relationship (Figs. 2 and 3). This implies that reducing the DLP value by adjusting the scan length and CTDI_vol_ can protect the thyroid from harmful radiation during the scan. However, the top priority remains optimising scan parameters and accurately positioning the patient in the isocentre to obtain images that are suitable for clinical diagnosis.^26^

### LAR results

The received dose, age at the time of radiation exposure, and sex are the three factors determining thyroid cancer development and its mortality rate. The LAR for thyroid cancer is higher in children and females than in adults and males.^16^ Moreover, a higher received dose increases the LAR of thyroid cancer and the related death rate. On the other hand, the risk decreases significantly with the increasing age of the patient.

The analysis of the LAR calculation revealed a significant difference between the two groups (*P* = 0.001, as shown in Table 5). The mean LAR for females was almost four times higher than that for males. However, both groups exhibited a decline in this risk with age, which is consistent with previous research (Fig. 3). The graph is split into three sections. For female patients, the maximum LAR for thyroid cancer occurs at approximately 4.7 per 100,000 patients with a received dose of 2.7 mGy at the age of 20 years. The LAR decreases with age, and the cancer risk falls to below 0.5 per 100,000 patients during the patient's lifetime. For those over 50 years old, the LAR value is zero, which ensures the safety of head CT scans in this group. The decreased LAR with age is linked to lower radiation sensitivity in older patients compared to younger ones.

The findings of this study are consistent with those of previous research. In males, the highest likelihood of LAR occurred at 20 years of age, with approximately 1.3 cases per 100,000 patients for a received dose of 2.5 mGy. Afterwards, there was a gradual decline in the LAR, and at 40–50 years of age, the LAR and thyroid cancer mortality rates were both zero, as shown in Figure 3. This trend is similar to the findings of previous studies.^12^, ^15^, ^18^

In this study, the scan parameters were almost identical for both male and female groups. However, it was observed that the female group's thyroid glands were more sensitive than those of the male group. This finding is consistent with numerous previous studies, including those by Narendran et al.,^39^ Iglesias et al.,^40^ Han et al.^29^ and Omer et al.,^13^ all of which have demonstrated the property of higher radiosensitivity in females. These studies have also found that females are more susceptible to developing thyroid cancer than males when exposed to ionising radiation, which is consistent with the findings of a previous study.^40^ Due to the high radiosensitivity of thyroids, several methods have been proposed to reduce the thyroid dose and the LAR while maintaining image quality. These methods include organ‐based modulation and thyroid shielding.^41^, ^42^ However, it is important to evaluate the efficacy of the existing dose reduction methods, protocols and guidelines, as well as develop new methods.^43^ In conclusion, it is necessary to strike a balance between the benefits of high‐quality imaging modalities and the risks to radiosensitive organs, which calls for further research.

There were several limitations to this study. Firstly, the sample size used was relatively small, even though it was statistically meaningful. To obtain more reliable results, a larger sample needs to be evaluated. Secondly, it should be noted that the phantoms used in CT‐expo were based on mathematical models that do not represent the real anatomy of the human body or reference phantoms for males and females. ICRU 103 recommends the use of computational phantoms based on CT scans or MRI images. Furthermore, CT‐Expo only includes two sizes of adult phantoms for dose estimation. Lastly, the estimation of LAR values in this study was based on the model recommended by BEIR VII, which has inherent uncertainty in the low dose range.

## Conclusion

This study aimed to determine and compare the thyroid organ dose, ED and LAR in routine head CT scans between the sexes. The LAR values were found to be significantly higher for females than for males and higher for younger patients than for older ones. This difference can be attributed to the anatomical differences between the two sexes. In addition, the current study aimed to estimate and compare thyroid organ dose, ED, and LAR in the males and females who had undergone routine head CT scans.

Performing CT procedures can increase thyroid organ dose and ED in both sexes. The organ dose of thyroid and ED increase with the increase of DLP, indicating that decreasing scan length can increase thyroid protection against harmful radiation exposure. A helical scan significantly increases thyroid dose in females and males compared with an axial scan. Consequently, an axial scan is recommended for head CT. There were three factors determining the LAR of the thyroid, including dose, age at the time of exposure and gender. The higher the radiation dose, the higher the LAR of cancer. The risk of radiation‐induced cancer increases with decreasing age; thus, children are more sensitive than adult patients. The LAR values were markedly higher for females than those for males.

## Funding Information

This work was supported by the Hormozgan University of Medical Sciences (Bandar Abbas, Iran) with the grant number of ‘980128’.

## Conflict of Interest

The authors declare no conflict of interest.

## Ethical Approval

This study was approved by a National Ethics Committee with the ethical committee consent number ‘HUMS.REC.1398.455’.

## Data Availability

The data that support the findings of this study are available from the corresponding author upon reasonable request.
